# Eletrocardiograma na Avaliação Pré-Operatória do Paciente de Baixo Risco: Evidências Atuais

**DOI:** 10.36660/abc.20230808

**Published:** 2024-02-16

**Authors:** Francisco José de Oliveira, Leonardo Rufino Garcia, Pedro Luciano Mellucci, Lenize da Silva Rodrigues, Matheus Bertanha

**Affiliations:** 1 Universidade Estadual Paulista Júlio Mesquita Filho Faculdade de Medicina Campus de Botucatu Botucatu SP Brasil Universidade Estadual Paulista Júlio de Mesquita Filho Faculdade de Medicina Campus de Botucatu, Botucatu, SP – Brasil

**Keywords:** Doenças Cardiovasculares/cirurgia, Assistência Perioperatória, Eletrocardiografia/métodos, Neoplasias/cirurgia, Medição de Risco

As doenças cardiovasculares representam a principal causa de mortalidade da população adulta, tanto no Brasil quanto globalmente. A mortalidade proporcional por essas doenças no Brasil é de aproximadamente 27%.^1^ As alterações cardiovasculares se tornam objeto de enorme preocupação para os pacientes que serão submetidos a procedimentos cirúrgicos, momento em que o organismo é exposto a alto nível de estresse metabólico e, dessa forma, complicações cardiovasculares podem se sobrepor com desfechos desfavoráveis. No mundo, estima-se que anualmente cerca de 900 mil pacientes submetidos a cirurgias evoluam com complicações cardíacas relacionadas diretamente ao ato cirúrgico.^2^ Neste contexto, o período perioperatório e, portanto, as alterações fisiológicas secundárias ao trauma cirúrgico são, isoladamente, um fator de risco para a manifestação de doenças cardiovasculares.

No Brasil, são realizadas entre 2 e 3 milhões de cirurgias por ano de acordo com o Departamento de Informática do Sistema Único de Saúde (DATASUS).^3^ A grandeza dos números reitera a importância da realização de um preparo pré-operatório pormenorizado para que sejam minimizados os riscos relativos ao procedimento cirúrgico, incluindo a avaliação cardiológica.

Dentre os exames complementares para avaliação pré-operatória cardiovascular, o eletrocardiograma (ECG) apresenta características de destaque, principalmente por ser um exame simples, não invasivo, barato, facilmente disponível e com interpretação relativamente simples por profissionais treinados. Por essas razões, o ECG se torna um importante instrumento diagnóstico na detecção de condições cardiovasculares em desenvolvimento ou já instaladas. A relevância do ECG como exame pré-operatório é reconhecida em grandes diretrizes aplicadas mundialmente, como a classificação de risco cirúrgico da *American Society of Anesthesiologists* (ASA),^4^ a *American College of Cardiology* / *American Heart Association* (ACC/AHA)^5^ e o *European Society of* Cardiology (ESC).^6^

A relação entre achados do ECG e os eventos adversos perioperatórios é classicamente descrita, incluindo anormalidades no traçado que podem predizer complicações cardíacas como infarto agudo do miocárdio e arritmias, ajudando na adoção de estratégias preventivas perioperatórias como a possibilidade de intervenções coronarianas precoces, controle medicamentoso e evitando eventos mais graves durante a anestesia.^7^

Importante ressaltar que a relevância do ECG varia de acordo com o tipo de cirurgia a ser realizada. Em procedimentos cirúrgicos de grande porte, como cirurgias cardiovasculares e para pacientes de alto risco cardiovascular, o ECG desempenha um papel crítico na avaliação pré-operatória, pois essas cirurgias estão mais associadas a eventos cardíacos adversos.^4-6^

Entretanto, o consenso da *European Society of* Cardiology recomenda a dispensa do ECG na avaliação pré-operatória cardiovascular de pacientes com idade inferior a 65 anos, sem fatores de risco cardiovasculares e que serão submetidos a procedimentos de baixo risco.^6,7^ Evidências atuais demonstram que a realização do ECG pré-operatório, nessas condições, não tem influência em reduzir a mortalidade perioperatória, além de ocasionar um aumento ao custo total do tratamento, não de forma direta, mas como um efeito em cadeia da sua sobreutilização, o que se apresenta em acordo com artigo *“Importância Prognóstica Do Eletrocardiograma Pré-Operatório Em Pacientes De Baixo Risco Submetidos À Intervenção Cirúrgica Sob Anestesia Geral”* publicado nesta edição.^8,9^

Deve-se ressaltar que a avaliação médica perioperatória deve ser realizada de forma individualizada e minuciosa, incluindo anamnese e exame clínico completos, não dispensando a realização de diversos exames complementares. Por vezes, o ECG pré-operatório ainda pode servir como padrão de comparação na ocorrência de alterações de traçado no perioperatório, em situações nas quais não exista uma alteração eletrocardiográfica prévia. Deve-se lembrar, contudo, que existem alterações do ECG que nem sempre se correlacionam com alterações cardíacas estruturais, sendo muitas vezes benignas em alguns subgrupos de pacientes, como os bloqueios atrioventriculares de 1° grau, bloqueios de condução intraventricular, bloqueios de ramo direito e bloqueios da divisão anterossuperior do ramo esquerdo, o que pode reforçar as conclusões dos autores já citados.^10^

Finalmente, a medicina baseada em evidências deve ser um baluarte na tomada de decisão e, nesse sentido, estudos com populações específicas podem apresentar limitações quando se propõe a tentativa de extrapolação para populações gerais.^6,9^ Porém, podem indicar o começo para a mudança de alguns paradigmas.
