# Supplementary material for: Eletrocardiograma na Avaliação Pré-Operatória do Paciente de Baixo Risco: Evidências Atuais
Source: Arq Bras Cardiol. 2024 Feb 16;121(1):e20230808. [Article in Portuguese] doi: 10.36660/abc.20230808 (PMC11081144; doi:10.36660/abc.20230808)
Supplement: Translation

# Translation: Electrocardiography in the Pre-Operative Assessment of Low-Risk Patients: Current Evidence

**Authors:** Francisco José de Oliveira, Leonardo Rufino Garcia, Pedro Luciano Mellucci, Lenize da Silva Rodrigues, Matheus Bertanha

**Affiliations:** 1 Botucatu SP Brazil Universidade Estadual Paulista Júlio de Mesquita Filho Faculdade de Medicina Campus de Botucatu, Botucatu, SP – Brazil

**Keywords:** Cardiovascular Diseases/surgery, Perioperative Care, Electrocardiography/methods, Neoplasms//surgery, Risk Assessment

Cardiovascular diseases represent the main cause of mortality in the adult population, both in Brazil and globally. The proportional mortality from these diseases in Brazil is approximately 27%.^1^ Cardiovascular alterations become the cause of great concern for patients who will undergo surgical procedures, a time when the body is exposed to a high level of metabolic stress and, therefore, cardiovascular complications can overlap with unfavorable outcomes. Worldwide, it is estimated that annually, approximately 900,000 patients undergoing surgery develop cardiac complications directly related to the surgery.^2^ In this context, the perioperative period and, therefore, physiological changes secondary to surgical trauma are a more important risk factor for the manifestation of cardiovascular diseases.

In Brazil, between 2 and 3 million surgeries are performed per year, according to the Department of Informatics of the Unified Health System (DATASUS).^3^ The magnitude of the data reiterates the importance of carrying out detailed pre-operative preparation so that the risks related to the surgical procedure are minimized, including cardiological evaluation.

Among the complementary tests for pre-operative cardiovascular assessment, the electrocardiogram (ECG) has outstanding characteristics, mainly because it is a simple, non-invasive, inexpensive, easily available test with relatively simple interpretation by trained professionals. For these reasons, the ECG becomes an important diagnostic tool in detecting developing or already established cardiovascular conditions. The relevance of the ECG as a pre-operative exam is recognized in major guidelines applied worldwide, such as the surgical risk classification of the American Society of Anesthesiologists (ASA),^4^ the American College of Cardiology / American Heart Association (ACC/AHA)^5^ and the European Society of Cardiology (ESC).^6^

The relationship between ECG findings and perioperative adverse events is classically described, including abnormalities in the tracing that can predict cardiac complications such as acute myocardial infarction and arrhythmias, helping in the adoption of preventive perioperative strategies such as early coronary interventions, medication control, and avoiding more serious events during anesthesia.^7^

It is important to highlight that the relevance of the ECG varies according to the type of surgery to be performed. In major surgical procedures, such as cardiovascular surgeries and for patients at high cardiovascular risk, the ECG plays a critical role in the pre-operative assessment, as these surgeries are more frequently associated with adverse cardiac events.^4-6^

However, the consensus of the European Society of Cardiology recommends that ECG should not be used in the pre-operative cardiovascular assessment of patients under the age of 65, without cardiovascular risk factors, and who will undergo low-risk procedures.^6,7^ Current evidence demonstrates that performing a pre-operative ECG under these conditions does not influence reducing perioperative mortality, in addition to causing an increase in the total cost of treatment, not directly, but as a chain effect of its overutilization, which agrees with the article “Prognostic Importance of Pre-Operative Electrocardiogram in Low-Risk Patients Undergoing Surgical Intervention Under General Anesthesia” published in this journal.^8,9^

It should be noted that the pre-operative medical evaluation must be carried out in an individualized and meticulous manner, including a complete anamnesis and clinical examination, without excluding the need for various complementary exams. Sometimes, the pre-operative ECG can still serve as a standard parameter for comparison in the occurrence of perioperative tracing changes in situations in which there was no previous electrocardiographic change. It should be remembered, however, that there are ECG changes that do not always correlate with structural cardiac changes and are often benign in some subgroups of patients, such as 1st-degree atrioventricular blockages, intraventricular conduction blockages, right bundle branch block and block of the anterosuperior division of the left bundle branch, which may reinforce the conclusions of the authors previously mentioned.^10^

Finally, evidence-based medicine must be the foundation in decision-making, and, in this sense, studies with specific populations may present limitations when trying to extrapolate to general populations.^6,9^ However, they may indicate the beginning of some paradigm changes.
